# Evaluation of a Novel *FKS1* R1354H Mutation Associated with Caspofungin Resistance in *Candida auris* Using the CRISPR-Cas9 System

**DOI:** 10.3390/jof9050529

**Published:** 2023-04-29

**Authors:** Maiko Kiyohara, Taiga Miyazaki, Michiyo Okamoto, Tatsuro Hirayama, Koichi Makimura, Hiroji Chibana, Nana Nakada, Yuya Ito, Makoto Sumiyoshi, Nobuyuki Ashizawa, Kazuaki Takeda, Naoki Iwanaga, Takahiro Takazono, Koichi Izumikawa, Katsunori Yanagihara, Shigeru Kohno, Hiroshi Mukae

**Affiliations:** 1 Department of Respiratory Medicine, Nagasaki University Graduate School of Biomedical Sciences, Nagasaki 852-8501, Japan; crystalblood2009@gmail.com (M.K.); tatsuro_h_20@nagasaki-u.ac.jp (T.H.); n-nakada@nagasaki-u.ac.jp (N.N.); y-ito@nagasaki-u.ac.jp (Y.I.); n-ashizawa@nagasaki-u.ac.jp (N.A.); k-takeda@nagasaki-u.ac.jp (K.T.); niwanaga@nagasaki-u.ac.jp (N.I.); takahiro-takazono@nagasaki-u.ac.jp (T.T.); s-kohno@nagasaki-u.ac.jp (S.K.); hmukae@nagasaki-u.ac.jp (H.M.); 2 Division of Respirology, Rheumatology, Infectious Diseases, and Neurology, Department of Internal Medicine, Faculty of Medicine, University of Miyazaki, Miyazaki 889-1692, Japan; makoto_sumiyoshi@med.miyazaki-u.ac.jp; 3 Medical Mycology Research Center, Chiba University, Chiba 260-8673, Japan; m-sato_okamoto@chiba-u.jp (M.O.); chibana@faculty.chiba-u.jp (H.C.); 4 Department of Pharmacotherapeutics, Nagasaki University Graduate School of Biomedical Sciences, Nagasaki 852-8501, Japan; 5 Teikyo University Institute of Medical Mycology, Tokyo 192-0395, Japan; makimura@med.teikyo-u.ac.jp; 6 Department of Infectious Diseases, Nagasaki University Graduate School of Biomedical Sciences, Nagasaki 852-8501, Japan; koizumik@nagasaki-u.ac.jp; 7 Department of Laboratory Medicine, Nagasaki University Hospital, Nagasaki 852-8501, Japan; k-yanagi@nagasaki-u.ac.jp

**Keywords:** *Candida auris*, echinocandin, caspofungin, antifungal resistance, *FKS1*, CRISPR-Cas9

## Abstract

Outbreaks of invasive infections, with high mortality rates, caused by multidrug-resistant *Candida auris* have been reported worldwide. Although hotspot mutations in *FKS1* are an established cause of echinocandin resistance, the actual contribution of these mutations to echinocandin resistance remains unknown. Here, we sequenced the *FKS1* gene of a caspofungin-resistant clinical isolate (clade I) and identified a novel resistance mutation (G4061A inducing R1354H). We applied the clustered regularly interspaced short palindromic repeats (CRISPR)-Cas9 system to generate a recovered strain (H1354R) in which only this single nucleotide mutation was reverted to its wild-type sequence. We also generated mutant strains with only the R1354H mutation introduced into *C. auris* wild-type strains (clade I and II) and analyzed their antifungal susceptibility. Compared to their parental strains, the R1354H mutants exhibited a 4- to 16-fold increase in caspofungin minimum inhibitory concentration (MIC) while the H1354R reverted strain exhibited a 4-fold decrease in caspofungin MIC. In a mouse model of disseminated candidiasis, the in vivo therapeutic effect of caspofungin was more closely related to the *FKS1* R1354H mutation and the virulence of the strain than its in vitro MIC. The CRISPR-Cas9 system could thus aid in elucidating the mechanism underlying drug resistance in *C. auris*.

## 1. Introduction

*Candida auris* is an emerging yeast species that was first isolated from the ear canal of an elderly Japanese woman in 2009 [1]. *C. auris* possesses a strong ability to colonize environmental surfaces and human skin, causing invasive infections. Severe outbreaks associated with multidrug-resistant *C. auris* have been reported in healthcare facilities worldwide [2,3,4,5]. The global in-hospital mortality rate of invasive *C. auris* infections is 30–60% [6]. Recently, outbreaks of fatal *C. auris* infection occurred in coronavirus disease (COVID-19) patients being treated in intensive care units [7,8,9,10]. In these cases, antifungal drug resistance contributed to the high mortality associated with *C. auris* infections.

The major antifungal agents currently used to treat invasive candidiasis are limited to four classes: echinocandins (e.g., caspofungin), azoles (e.g., fluconazole), polyenes (e.g., amphotericin B), and fluorinated pyrimidine (flucytosine). The Centers for Disease Control and Prevention (CDC) reported that among the 323 *C. auris* clinical isolates identified in 2018, 90% were resistant to at least one class of antifungal agents and 30% were resistant to two or more classes [11]. In addition, an alarming increase in clinical cases involving antifungal-resistant *C. auris* infections was reported in the United States (476, 756, and 1471 cases in 2019, 2020, and 2021, respectively) [12]. In the global estimates, although regional and clade-specific variations exist [13,14,15], more than 90% of *C. auris* clinical isolates are resistant to fluconazole, 30–35% to amphotericin B, and 5–7% to echinocandins; multidrug resistance is observed in 41% of the isolates and pan-resistance (resistance to three antifungal classes) in 3–4% [16,17]. Echinocandins are the recommended first-line therapy for invasive candidiasis including *C. auris* infections. However, 37% of the 90 isolates detected in five facilities in India exhibited increased caspofungin MICs [18]. Chen et al. reported that resistance rates to caspofungin, anidulafungin, and micafungin in *C. auris* were 12.1% (101/838), 1.1% (9/840), and 0.8% (8/927), respectively, and almost all isolates resistant to caspofungin were from India, with a resistance rate of 23.6% (100/424) for Indian isolates and of 0.2% (1/414) for non-Indian isolates [19]. In a retrospective multicenter study of *C. auris* infections conducted in southern Asia and the US, the most common echinocandins used for treatment were caspofungin (40%) followed by anidulafungin (28%) and micafungin (15%), and the resistance rates of *C. auris* strains to caspofungin, anidulafungin, and micafungin were 16, 5, and 0%, respectively [20]. Furthermore, the number of *C. auris* cases resistant to echinocandins in the US tripled in 2021 compared with that in 2020 [12]. Since fluconazole and amphotericin B resistance is common in *C. auris* clinical isolates, the emergence of echinocandin-resistant strains poses a serious threat.

Echinocandins disrupt the integrity of the fungal cell wall by inhibiting the β-1-3-glucan synthase encoded by the *FKS* genes. In *Candida albicans*, the substitution of the amino acid serine at position 645 in the hotspot-1 region of *FKS1* produces the most pronounced echinocandin-resistant phenotype [21,22,23]. Similarly, in *C. auris*, S639F and S639P in the hotspot-1 of *FKS1* are associated with echinocandin resistance [24]. Few mutations have been identified that confer echinocandin resistance and are more reliable than the in vitro MIC for predicting the therapeutic failure of echinocandin in vivo [25]. Therefore, further elucidation of the *C. auris FKS1* genotype is necessary. It is unclear how each mutation contributes to drug resistance because clinical isolates usually harbor multiple mutations in the genome. Theoretically, comparing a parent strain to a mutant generated through the genetic manipulation of a single nucleotide is necessary to evaluate the effects of the corresponding mutation.

The construction of genetic variants of *C. auris* is challenging because the clustered regularly interspaced short palindromic repeats (CRISPR)-Cas9 genome editing technique designed for *C. albicans* is not effective for *C. auris* despite the expression constructs operating efficiently in *C. albicans*. Recently, an RNA-mediated CRISPR-Cas9 gene editing system for *C. auris* was established [26]; however, studies on the construction of single nucleotide substitutions are scarce [27].

In this study, we sequenced the *FKS* genes of *C. auris* clinical isolates and identified candidate mutations that may cause echinocandin resistance. We constructed a mutant strain containing a single candidate mutation from a *C. auris* wild-type strain using the RNA-mediated CRISPR-Cas9 gene editing system. In addition, we generated a recovered strain, in which only the candidate mutation was reversed to its wild-type sequence, from the clinical isolate. We evaluated the changes in MIC due to single nucleotide substitutions through in vitro antifungal susceptibility testing. Lastly, we studied the effects of the mutation on the fitness cost and in vivo response to caspofungin treatment using a mouse model of disseminated candidiasis. Genome editing technologies and genetic tools for *C. auris* are limited [26,27]. To the best of our knowledge, this is the first report of a single nucleotide substitution introduced in *C. auris FKS1* using the CRISPR-Cas9 genome editing technique.

## 2. Materials and Methods

### 2.1. Strains and Culture Conditions

The *C. auris* strains used in this study are listed in Table 1. The type strain of clade II *C. auris* (JCM15448: first isolated from aural discharge in Japan in 2009) was provided by Teikyo University [1]. Two clinical isolates of clade I *C. auris* (NCPF8971 and NCPF8985) were isolated from wound swabs in the UK in 2015 and 2016, respectively, and obtained from the National Collection of Pathogenic Fungi [5]. The *C. auris* strains were routinely propagated in yeast peptone dextrose (YPD) medium (1% yeast extract, 2% peptone, 2% dextrose). The genetically modified strains were selected by culturing them in YPD agar medium containing 100 µg/mL nourseothricin (Jena Bioscience GmbH, Jena, Germany).

### 2.2. Sequence Analysis of the C. auris FKS Genes

Genomic DNA was extracted from each *C. auris* strain using a DNA extraction kit (Qiagen, Hilden, Germany). Primers for the amplification and sequencing of the *FKS1* region were designed as previously described [24,26] and are listed in Table 2. PCR was performed using KOD One PCR Master Mix (TOYOBO, Osaka, Japan) and a T100 Thermal Cycler (Bio-Rad Laboratories, Inc., Hercules, CA, USA). DNA sequencing was carried out by Macrogen Japan Corp. (Tokyo, Japan), and nucleotide sequences of the *FKS1* region in the clinical isolates were compared to the reference sequences of the *C. auris* clade I strain (GenBank accession number B9J08_000964).

### 2.3. Genetic Manipulation

We used the CRISPR-Cas9 technique [26] to introduce single nucleotide substitutions into the *C. auris* strains. A schematic illustration of the procedure is shown in Supplementary Figure S1. The repair cassette containing the nourseothricin-resistant (*NatR*) marker was obtained through fusion PCR using three DNA fragments with overlapping sequences: an approximately 2.7 kb DNA fragment from the genomic DNA of strain JCM15448 or NCPF8985 containing the *FKS1* hotspot-2 region was amplified using the primers Cau1ST-F and Cau1ST-R; an approximately 1.3 kb DNA fragment containing the *NatR* marker from the plasmid pNAT [28] was amplified using the primers NatR-F and NatR-R; and an approximately 1.0 kb DNA fragment containing the 5′ region (upstream of hotspot-2) of the *FKS1* open reading frame from the parental strain genomic DNA was amplified using the primers Cau3RD-F and Cau3RD-R. These three DNA fragments were purified and used for fusion PCR to yield an approximately 2.5 kb repair cassette.

The CRISPR RNAs (crRNAs) were developed using the Alt-R CRISPR HDR Design Tool (Integrated DNA Technologies, Coralville, IA, USA). We designed two types of crRNAs containing the closest 20 bp long upstream and downstream homologous sequences across the target base of *FKS1*: RZVT5652-AD (located at 4031–4050, ACTTGTCTCCTGCGATCGAC) and RZVT5652-AB (located at 4070–4089, TGTCCATTTTCATCGTCTTC). The designed crRNAs (4 μM) and universal trans-activating crRNAs (4 μM) were incubated at 95 °C for 5 min to create guide RNAs, which were then mixed with Cas9 nuclease (4 μM) in a 1.2:1 ratio to create RNA–protein complexes.

Two RNA–protein complexes (4 µL each) were mixed with 1 µg (5 μL) of purified repaired DNA. The mixture was combined with a pellet of *C. auris* cells adjusted as previously reported and transformed using electroporation (Gene Pulser Xcell, Bio-Rad, Hercules, CA, USA) [26]. The transformants were cultured in a YPD broth at 30 °C with shaking at 120 rpm for 4 h and were further seeded on a YPD agar medium containing nourseothricin. The plates were incubated at 37 °C for 2 d. DNA extracted from the colonies was sequenced to confirm that it contained the desired substitution without off-target mutations in the *FKS1* region. The transformants were stored at −80 °C.

### 2.4. In Vitro Growth Curve and Antifungal Susceptibility Testing

*C. auris* cells in their exponential growth phase were suspended in YPD broth and adjusted to an optical density of 0.1 at 600 nm (OD_600_), corresponding to approximately 6.5 × 10^5^ CFU/mL. The cells were incubated at 37 °C with shaking at 250 rpm, and OD_600_ was measured at 0, 2, 4, 6, 8, 10, 12, 24, and 30 h. The generation time was calculated as previously described [29]. The experiment was performed in triplicate and repeated twice.

In vitro antifungal susceptibility was determined using the Sensititre YeastOne colorimetric microdilution panel (Trek Diagnostic Systems, Ltd., East Grinstead, UK), following the manufacturer’s instructions. The results were visually checked and assessed by two investigators. Antifungal susceptibility tests were repeated at least twice to ensure reproducibility.

### 2.5. In Vivo Experiments

All animal experiments in this study were performed in accordance with the Guide for the Care and Use of Laboratory Animals [30] and all institutional regulations and guidelines for animal experimentation following review and approval by the Institutional Animal Care and Use Committee at the Nagasaki University (approval number 1906121536).

A well-established neutropenic mouse model of disseminated candidiasis was used in this study [24,31,32]. Female, 8-week-old, BALB/c mice (Charles River Laboratories Japan, Yokohama, Japan) weighing 19–23 g were immunosuppressed by subcutaneously injecting them with 200 µL of cyclophosphamide (Sigma–Aldrich Japan, Tokyo, Japan), as described previously [31]. Four days prior to *C. auris* infection, cyclophosphamide was administered at a dose of 150 mg/kg, and one day before infection and two and five days after infection (days −1, 2, and 5), cyclophosphamide was administered at a dose of 100 mg/kg. In the survival study, cyclophosphamide was administered every 3 d until day 14.

For inoculum preparation, *C. auris* cells in the exponential growth phase, grown in YPD broth, were harvested and washed twice with saline. The cells were then resuspended in saline and adjusted to 5 × 10^7^ CFU/mL and 2.5 × 10^6^ CFU/mL for evaluating mouse survival and the fungal burden in infected mouse organs, respectively. The immunosuppressed mice (10–11 mice per group) were infected with 200 µL of *C. auris* inoculum (1 × 10^7^ CFU/mouse) intravenously on day 0. The infected mice were randomly assigned to the caspofungin group and control group. The mice in the caspofungin group were intraperitoneally treated with 0.76 mg/kg caspofungin (MSD K.K., Tokyo, Japan), which corresponded to the human therapeutic dose, or 7.6 mg/kg caspofungin, which corresponded to the high dose. Caspofungin treatment was initiated 2 h after inoculation with *C. auris* cells and continued once daily until day 14. The mice in the control group were treated with saline in the same manner. The survival of the mice was monitored until day 21.

To evaluate the fungal burden in target organs, eight groups of immunosuppressed mice (6 or 7 mice per group) were infected with 200 µL of *C. auris* inoculum (5 × 10^5^ CFU/mouse) intravenously on day 0. The mice were treated with caspofungin, as described earlier until day 5, and euthanized through carbon dioxide overexposure on day 6. The heart and bilateral kidneys were excised, and the organs were homogenized in saline. Serial dilutions of organ homogenates were plated on YPD agar medium and incubated at 37 °C for 2 d. CFU counts were performed, and the CFU values per organ were calculated. All mouse experiments were performed at least twice to ensure reproducibility.

### 2.6. Statistical Analysis

The Kaplan–Meier survival curves were compared via a log-rank test, and *p* < 0.05 was considered statistically significant. Differences in the fungal burden in mouse organs were analyzed using the Mann–Whitney U test with Bonferroni correction, and *p* < 0.0125 was considered statistically significant. All analyses were conducted using Prism9 Version 9.3.1 (GraphPad Software, San Diego, CA, USA).

## 3. Results

### 3.1. R1354H Mutation in FKS1 Causes Increased Echinocandin MICs in C. auris

DNA sequencing of the hotspot-1 and hotspot-2 regions of *FKS1* was performed for the *C. auris* strains JCM15448, NCPF8971, and NCPF8985. Although the known mutations (S639F, S639P, F635Y, and F635L) associated with echinocandin resistance were not present in these strains, the R1354H (G4061A) mutation was found in the *FKS1* hotspot-2 region in the caspofungin-resistant strain NCPF8985 but not in the echinocandin-susceptible strains JCM15448 and NCPF8971.

To examine whether the R1354H mutation is involved in echinocandin resistance, this mutation was introduced into the JCM15448 and NCPF8971 strains to generate the mutants JCM15448-MT and NCPF8971-MT, respectively. In contrast, the R1354H mutation in NCPF8985 was reversed to the wild-type sequence, yielding NCPF8985-RV. The in vitro antifungal susceptibilities of these six strains are shown in Table 3. The introduction of the R1354H mutation resulted in increased MICs for all echinocandins tested in this study, including a 4-fold increase for the JCM15448 strain background and a 4- to 16-fold increase for the NCPF8971 strain background. Conversely, NCPF8985-RV exhibited a 4-fold decrease in all echinocandin MICs compared to the echinocandin MICs for NCPF8985. These 4-fold changes in MIC were consistently reproduced in three independent tests. These results indicate that the R1354H mutation is associated with elevated echinocandin MICs in *C. auris*. The R1354H mutation did not affect the in vitro growth capacity (Supplementary Figure S2) and susceptibility of *C. auris* to azoles, amphotericin B, and flucytosine (Table 3).

### 3.2. Efficacy of Caspofungin Treatment in Mice Infected with C. auris Strains Harboring the FKS1 R1354H Mutation

**Figure 1:**
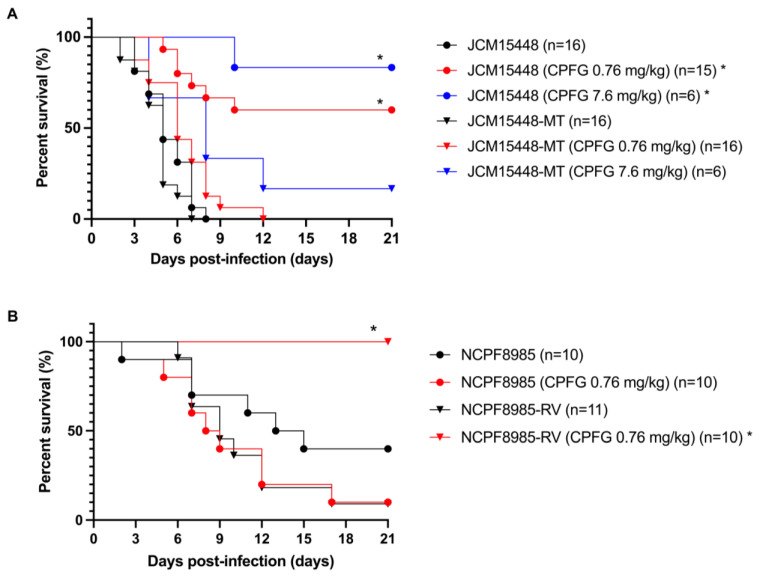
Kaplan–Meier survival curves of mice infected with *C. auris* strains. Immunosuppressed mice were intravenously injected with *C. auris* inoculum (1 × 10^7^ CFU/mouse). (**A**) The mice were infected with JCM15448 (parental strain, R1354) or JCM15448-MT (FKS1c.4061G>A::NatR, R1354H), and they were intraperitoneally treated with caspofungin (0.76 or 7.6 mg/kg) or saline. (**B**) The mice were infected with NCPF8985 (parental strain, H1354) or NCPF8985-RV (FKS1c.4061A>G::NatR, H1354R), and they were intraperitoneally treated with caspofungin (0.76 mg/kg) or saline. Treatment was initiated 2 h after inoculation with *C. auris* cells and continued once daily until day 14. Mouse survival was monitored until day 21. The data were analyzed using the log-rank test. * *p* < 0.0001 (vs. control groups treated with saline). The representative data of three independent experiments are shown.

To examine the effects of the R1354H mutation on the in vivo susceptibility of *C. auris* to caspofungin, immunocompromised mice were intravenously infected with either JCM15448 or JCM15448-MT and further treated with caspofungin or saline (Figure 1A). Without antifungal treatment, the mice infected with JCM15448 and JCM15448-MT exhibited similar survival curves and 100% mortality within 8 days [median survival time (MST) was 5 days in both groups]. Caspofungin treatment at the standard dose (0.76 mg/kg) was highly effective for mice infected with JCM15448 but had minor effects on mice infected with JCM15448-MT. The high-dose caspofungin treatment (7.6 mg/kg) tended to improve the survival of mice in both groups compared to the standard-dose treatment, although the differences were not statistically significant. A comparison of the survival curves of mice infected with JCM15448 and NCPF8985 showed that NCPF8985 appeared to be less virulent than JCM15448 (Figure 1A,B). Caspofungin treatment at the standard dose was effective in mice infected with NCPF8985-RV but not in those infected with NCPF8985 (Figure 1B).

**Figure 2:**
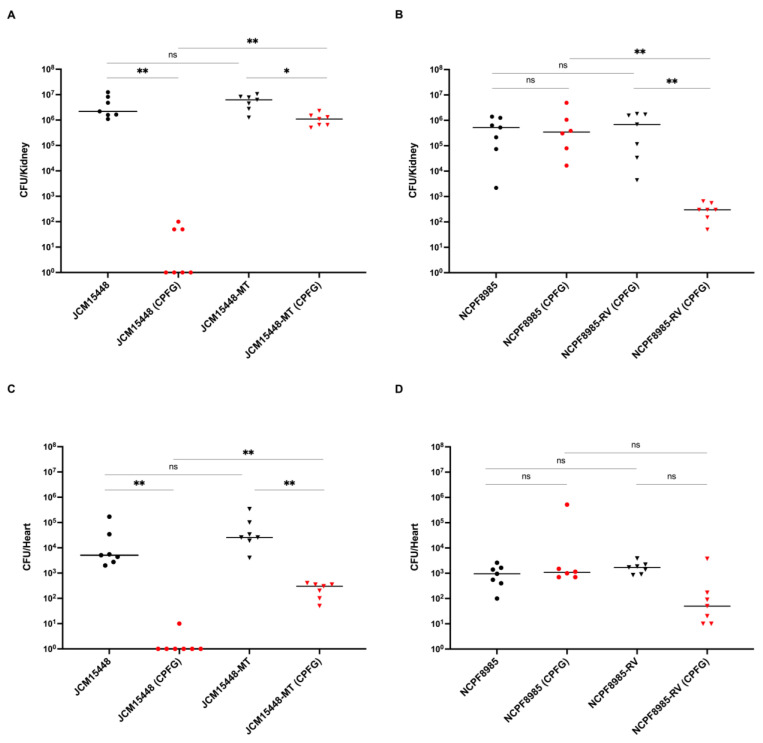
Fungal burden in the target organs of mice infected with *C. auris* strains. Immunosuppressed mice (6 or 7 mice per group) were intravenously infected with *C. auris* inoculum (5 × 10^5^ CFU/mouse) on day 0. The mice were intraperitoneally treated with caspofungin (0.76 mg/kg) or saline. Treatment was initiated 2 h after inoculation with *C. auris* cells and continued once daily until day 5. The mice were euthanized on day 6, and target organs were excised. Serial dilutions of organ homogenates were plated on YPD agar medium. Plates were incubated for 2 d, and CFU values per organ were calculated. CFU counts recovered from the kidney (**A**,**B**) and heart (**C**,**D**) are indicated for individual mice in the scatter plots. The data were analyzed using the Mann–Whitney U test and adjusted by Bonferroni correction, and *p* < 0.0125 was considered statistically significant. The representative data of three independent experiments are shown. The bars represent medians. * *p* < 0.0125, ** *p* < 0.0012. ns, not significant; black dots, parent strain treated with saline; red dots, parent strain treated with caspofungin; black triangle, edited strain treated with saline; red triangle, edited strain treated with caspofungin.

We also examined the fungal organ burden using a mouse model of disseminated candidiasis to evaluate the virulence of the *C. auris* strains and the therapeutic efficacy of caspofungin. Immunocompromised mice with systemic *C. auris* infections were euthanized on day 6, and the target organs (kidney and heart) were excised. The number of *C. auris* cells recovered from the kidney (Figure 2A,B) was higher than that recovered from the heart (Figure 2C,D). The fungal burden in these organs was comparable between the mice infected with JCM15448 and JCM15448-MT (Figure 2A,C) and between those infected with NCPF8985 and NCPF8985-RV (Figure 2B,D), suggesting that arginine or histidine at position 1354 in *FKS1* does not affect the virulence of *C. auris* in mice. Caspofungin treatment led to a marked reduction in the fungal burden in all target organs in the mice infected with JCM15448 and NCPF8985-RV but showed little or no efficacy in the mice infected with JCM15448-MT and NCPF8985.

Overall, the results of the fungal burden assay were consistent with those of the survival assay, suggesting that infection with *C. auris* harboring the *FKS1* R1354H mutation is refractory to treatment with caspofungin in vivo.

## 4. Discussion

If the echinocandin-resistant clinical isolates had amino acid mutations in the *FKS1* hotspot regions, those mutations were considered responsible for the echinocandin-resistant phenotypes. The R1354H substitution in *C. auris* likely corresponds to the previously reported R1361G mutation in *Candida krusei* (caspofungin MIC: 32 μg/mL) and the R1361H mutation in *Candida albicans* (caspofungin MIC: 2 μg/mL) [21,22]. Recently, a *C. auris* R1354S (C4060A) mutant (caspofungin MIC: 16 μg/mL) was isolated in India [25]. These results suggest that the corresponding mutations may be involved in caspofungin resistance as well. However, the extent to which such mutations contribute to caspofungin resistance cannot be determined because the possible involvement of other mutations or resistance mechanisms has not been ruled out. In this study, we analyzed the effect of a single amino acid substitution on antifungal susceptibility using a bidirectional approach. We found that the introduction of the R1354H mutation into a wild-type strain caused caspofungin resistance. Moreover, the R1354H mutation was able to revert the resistant strain to its wild-type genotype (H1354R), thereby restoring caspofungin susceptibility. Furthermore, we established a mouse model of disseminated candidiasis, analyzed the survival curve and fungal burden in the target organs, and found that the *C. auris* R1354H mutants were resistant to caspofungin in vivo.

Although *C. auris*-specific susceptibility breakpoints have not been established, the CDC published the following provisional MIC breakpoints: micafungin ≥ 4 µg/mL, caspofungin ≥ 2 µg/mL, and anidulafungin ≥ 4 µg/mL (https://www.cdc.gov/fungal/candida-auris/c-auris-antifungal.html, accessed on 29 May 2020). The strains examined in this study exhibited varying base MICs for different antifungal agents. The amino acid substitution at position 1354 (R1354H and H1354R) consistently led to an approximately 4-fold increase or decrease in the MIC, and whether they exceeded the breakpoints was dependent on the original MIC. Intriguingly, although JCM15448-MT (caspofungin MIC: 0.12 µg/mL) was classified as susceptible using the tentative breakpoints described above, it was refractory to caspofungin treatment in infected mice. Moreover, NCPF8985-RV (caspofungin MIC: 2 µg/mL) remained resistant to caspofungin in vitro but became sensitive to caspofungin treatment in infected mice. The results in the present study support the idea that the *FKS1* genotype is a more reliable predictor of in vivo treatment response than the in vitro MIC, as previously suggested [25]. However, caution must be exercised when comparing treatment effects in strains with different virulence backgrounds, as illustrated by the lower virulence of NCPF8985 compared with JCM15448.

Several limitations of the present study should be mentioned. First, we did not include a pair of the strains NCPF8971 and NCPF8971-MT in mouse experiments because their echinocandin susceptibility phenotypes in vitro were similar to those of the pair of JCM15448 and JCM15448-MT. Second, the therapeutic effects of micafungin and anidulafungin have not been evaluated in mice infected with the *C. auris* strains. Third, we did not analyze the effects of the *FKS1* R1354H mutation on cell wall composition and glucan synthase activity. However, when comparing the in vitro growth capacity and virulence in mice with the same strain backgrounds, namely, JCM15448 vs. JCM15448-MT and NCPF8985 vs. NCPF8985-RV, the R1354H mutation did not affect the fitness cost in *C. auris*. Hence, this mutation may be present in clinical isolates that are classified as echinocandin-susceptible in vitro. Consequently, we should be aware of stealthy echinocandin-resistant *C. auris* strains, such as the R1354H mutants, because they cannot be detected by in vitro susceptibility screening. Generally, *C. auris* isolates detected in Japan and Korea are clade II strains isolated from patients with non-invasive infections and are susceptible to echinocandins in vitro [33]. However, similar to NCPF8985, JCM15448-MT was virulent and caspofungin-resistant in vivo. This indicates that clade II strains that have not caused outbreaks could also pose a serious risk to public health. Moreover, Du et al. reported that clade II strains have gradually become resistant to antifungal agents in recent years [34].

In this study, the expression-free CRISPR-Cas9 genome editing system was successfully applied to *C. auris* to demonstrate the effects of specific gene mutations on drug susceptibility and pathogenicity. This method should be utilized in the evaluation of other clinical isolates including clade III and clade IV strains in the future. *C. auris* has been placed in the “urgent” category on the CDC’s Priority List for drug discovery against antimicrobial-resistant pathogens. Very recently, the CDC reported the worsening spread of *C. auris* in the US as an increasing threat to public health [12]. The expression-free CRISPR-Cas9 genome editing system can be useful in developing drug targets and further elucidating the mechanism underlying drug resistance.

## Figures and Tables

**Table 1:** *C. auris* strains used in this study.

Strain	Clade *	Genotype or Description	Reference or Source
JCM15448	Ⅱ	Type strain, Parental strain (R1354)	Teikyo University [1]
NPCF8971	Ⅰ	Parental strain (R1354)	National Collection of Pathogenic Fungi [5]
NPCF8985	Ⅰ	Parental strain (H1354)	National Collection of Pathogenic Fungi [5]
JCM15448-MT	Ⅱ	FKS1c.4061G>A::NatR (R1354H)	This study
NPCF8971-MT	Ⅰ	FKS1c.4061G>A::NatR (R1354H)	This study
NPCF8985-RV	Ⅰ	FKS1c.4061A>G::NatR (H1354R)	This study

* Clade I, South Asia; Clade II, East Asia.

**Table 2:** Primers used in this study.

For *FKS1* Sequencing	
Primer	Sequence (5′-3′)
CauFKS1-F	ATGTCTTACGATAACAATC
CauFKS1-R	TTAGAATGCCTTTGTAGTATAG
CauFKS1-F653	GAAGTGGTTTTTCGCCTCG
CauFKS1-F1256	AGAGATACATGAGATTGGGTG
CauFKS1-F1863	TCTTTGGGTCACTGTGTTTG
CauFKS1-F2446	CGTATCAGTTTCTTTGCTCAG
CauFKS1-F3048	CGCCGAGTTTTTGTTGAGAG
CauFKS1-F3674	GTATGACTGCCATGTTGAGAG
CauFKS1-F4241	CAGACTTGACCGTTGGTGGTG
CauFKS1-F4878	CTGTCTTGGAATGGCTTGTTG
CauFKS1-R178	AGTTCTGGTAACCGTCCATG
CauFKS1-F67	GAGTATTACCAACAGGGCG
CauFKS1-F5303	TAGACAGATGGCACTCCACC
For genetic manipulation	
Primer	Sequence (5′-3′)
Cau1ST-F	CGCCGAGTTTTTGTTGAGAG
Cau1ST-R	CGGCGGGGACGAGGCAAGCTTACAGCTTGTAGAATTCTCGAAT
NatR-F	TATTATTCTATATTGGTTTGAATTAAGCTTGCCTCGTCCCCGCCGGGTCA
NatR-R	GCTTGTAGAATTCTCGAATAATGTAGACACTGGATGGCGGCGTTAGTATC
Cau3RD-F	GATACTAACGCCGCCATCCAGTGTCTACATTATTCGAGAATTCTACAAGCTG
Cau3RD-R	GGTAGCATTCAAGAACCAAGAC

**Table 3:** Antifungal susceptibilities of *C. auris* strains.

Strain	MIC (μg/mL)								
	AFG	MFG	CAS	5FC	POS	VRC	ITC	FLC	AMB
JCM15448	0.12	0.06	0.03	<0.06	0.015	0.015	0.03	4	0.5
JCM15448-MT	0.5	0.25	0.12	<0.06	0.015	0.015	0.03	2	0.5
NCPF8971	0.12	0.12	0.12	<0.06	<0.008	0.25	0.06	128	1
NCPF8971-MT	1	0.5	2	<0.06	<0.008	0.25	0.06	>256	1
NCPF8985	2	2	8	>64	>8	>8	>16	>256	1
NCPF8985-RV	0.5	0.5	2	>64	>8	>8	>16	>256	1

AFG, anidulafungin; MFG, micafungin; CAS, caspofungin; 5FC, 5-flucytosine; POS, posaconazole; VRC, voriconazole; ITC, itraconazole; FLC, fluconazole; AMB, amphotericin B.

## Data Availability

All data generated or analyzed during this study are included in this published article and its Supplementary Information files.

## Supplementary Materials

The following supporting information can be downloaded at: https://www.mdpi.com/article/10.3390/jof9050529/s1, Figure S1: Schematic diagram of genetic manipulation using the CRISPR-Cas9 system in *Candida auris*; Figure S2: In vitro growth curves of the *C. auris* strains.
