# Supplementary material for: Evaluation of a Novel FKS1 R1354H Mutation Associated with Caspofungin Resistance in Candida auris Using the CRISPR-Cas9 System
Source: J Fungi (Basel). 2023 Apr 29;9(5):529. doi: 10.3390/jof9050529 (PMC10219442; doi:10.3390/jof9050529)
Supplement: Supplementary file 1 [file jof-09-00529-s001.zip › Supplementary Figure S1.pdf]

## Evaluation of a Novel *FKS1* R1354H Mutation Associated with Caspofungin Resistance in *Candida auris* Using the CRISPR-Cas9 System

Kiyohara M, et al.

### Supplemental Figure S1

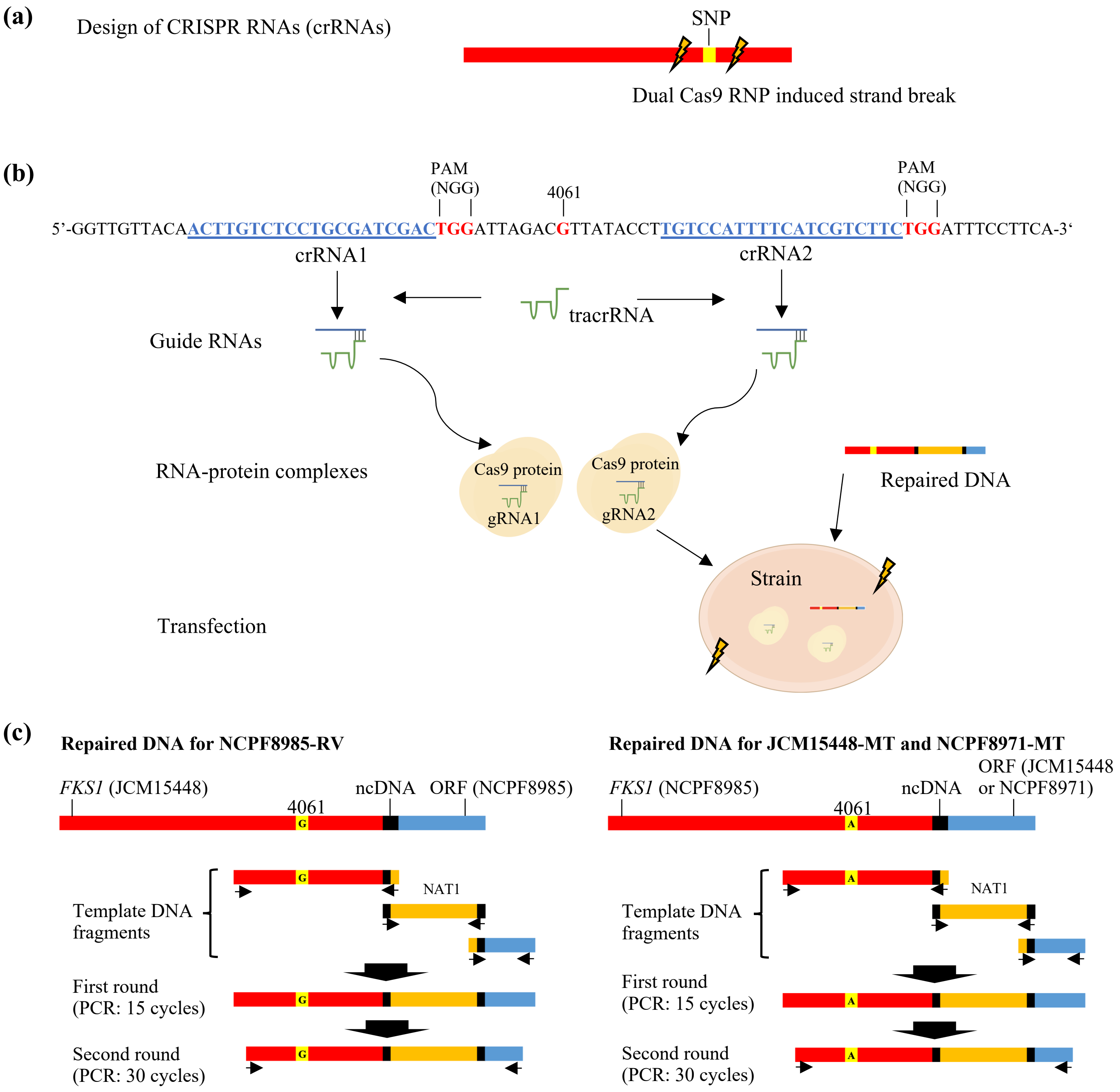


**Supplemental Figure S1. Schematic diagram of genetic manipulation using the CRISPR-Cas9 system in *Candida auris*.** (a) Two types of CRISPR RNAs (crRNAs) closest to the point mutation were generated using the Alt-R CRISPR HDR Design Tool. (b) The crRNAs were incubated with tracrRNA and guide RNAs, respectively, to form RNA-Cas 9 protein complexes (RNPs). RNPs and repaired DNA constructs described below were introduced into cells by electroporation. (c) The repaired DNA constructs containing the NAT gene between *FKS1* and the adjacent open reading frame (ORF) were generated. First, three PCR fragments (template DNA fragments) were prepared and purified, and then first round PCR (15 cycles) was performed with only the three fragments. Next, second round PCR (30 cycles) was performed using the first round PCR product as a template and a primer pair listed in Table 2 to generate repaired DNA.
