# Supplementary material for: Evaluation of a Novel FKS1 R1354H Mutation Associated with Caspofungin Resistance in Candida auris Using the CRISPR-Cas9 System
Source: J Fungi (Basel). 2023 Apr 29;9(5):529. doi: 10.3390/jof9050529 (PMC10219442; doi:10.3390/jof9050529)
Supplement: Supplementary file 1 [file jof-09-00529-s001.zip › Supplementary Figure S2.pdf]

Supplementary Figure S2. ***In vitro*** **growth curves of the** ***C. auris*** **strains**

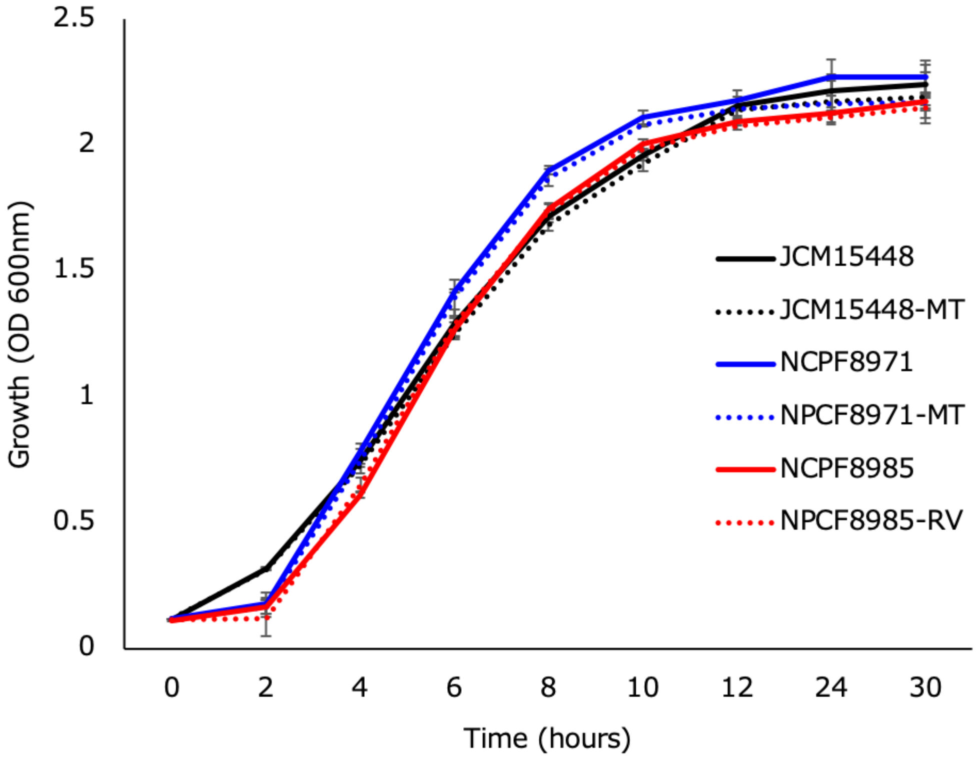

Strains	Doubling time (h)
JCM15448	1.72 ± 0.01
JCM15448-MT	1.77 ± 0.01
NCPF8971	1.67 ± 0.00
NPCF89871-MT	1.69 ± 0.03
NCPF8985	1.71 ± 0.02
NPCF8985-RV	1.75 ± 0.03
